# An analysis of 13 patients with perforated gastric carcinoma: A surgeon's nightmare?

**DOI:** 10.1186/1749-7922-3-17

**Published:** 2008-05-10

**Authors:** Cetin Kotan, Aziz Sumer, Murat Baser, Remzi Kızıltan, M Ali Carparlar

**Affiliations:** 1 Department of Surgery, Faculty of Medicine, Yüzüncü Yil University, Van, Turkey; 2 Department of Surgery, Kaş State Hospital, Antalya, Turkey

## Abstract

**Background and Objectives:**

Perforation is a rare complication of gastric carcinoma and generally not diagnosed preoperatively. To clarify the clinicopathologic characteristics of patients with this condition we reviewed 13 cases of gastric cancer perforation who required emergency surgery.

**Methods:**

A total of 13 patients with gastric cancer perforation were retrospectively reviewed. The clinicopathological features including tumor stage and survival and also the type of treatment were analyzed and compared to literature data.

**Results:**

There were 13 patients (10 males and 3 females) with a mean age of 59.0 ± 9.56 years. The incidence of perforated gastric cancer was 9.6% among gastric carcinoma and 4.2% of all gastric perforation cases. The perforation was more frequently in stage III–IV (2–10), but one case of stage II (T3N0M0) gastric cancer was also observed. None of the patients had curative resection or radical lymph-node dissection. Six (46%) patients were treated by palliative, local surgery. Emergency gastrectomy were performed in 7 (54%) patients. Overall 30-day mortality rate was % 46. The overall survival time was 128.2 ± 184.8 days for all patients, it was 52.8 ± 52.9 days for locally treated group, and 192.9 ± 235.4 days for patients who underwent resectional surgery. The difference between the treatment groups was not significant

**Conclusion:**

Perforation usually occurs in advanced stages of gastric cancer. These patients had a poor prognosis because of the presence of advanced cancer.

## Introduction

Oncologic emergencies in patients with gastric cancer include perforation and major bleeding. These complications require emergency treatment and have a high mortality rate[1,2]. Perforated gastric cancer (PGC) is a rare condition with a reported incidence of 0.3–3.9%, and generally present with histories and symptoms that do not differ obviously from those of benign gastric perforation [1-5]. In most instances, gastric carcinoma is not suspected as the cause of perforation prior to emergency laparotomy [6]. Even during surgery the gastric ulcer is often difficult to be characterized as benign or malignant by the surgeon, especially when a frozen section is unavailable [6,7].

Since PGC is clinically characterized by generalized peritonitis and frequently occurs at an advanced stage of the disease, it usually results in a poor outcome, and long-term survival seems to be rare [4,8].

The treatment should aim to manage both the emergency condition of peritonitis and the oncologic technical aspects of surgery.

Factors influencing the surgical results and recommended treatment strategies are unclear.

The aim of this study is to address these questions by analyzing a series of 13 patients who were treated for perforated, advanced stage adenocarcinoma of the stomach over the last 8 years.

## Patients and methods

This work is a retrospectively descriptive study of perforated gastric cancer. We reviewed the medical records of 13 patients with PGC who had undergone surgical treatment in the Yuzuncu Yıl University, Medical Faculty Hospital. The clinicopathological features of all patients were analyzed on the basis of their medical records. Age, sex, preoperative diagnosis, surgical procedure, location of perforation, depth of gastric wall invasion, presence of lymph node metastasis, presence of distant metastases (liver metastases or intraperitoneal secondary deposits), type of surgery, degree of lymph node dissection, type of resection-complete (R0) or incomplete resection (R1 or R2) – stage, and outcome of the patients were examined. The study also assessed the impact of the preoperative SIRS/sepsis on hospital mortality rate. Sepsis parameters were defined according to the International Sepsis Definitions Conference diagnostic criteria for sepsis [9].

These clinicopathologic findings were determined on the basis of the general rules of American Joint Commitee on Cancer (AJCC) [10].

Kaplan-Meir statistical analyses method was employed for ..Survival statistical analysis.

## Results

The clinicopathological features of the 13 patients – 10 males and 3 females, aged from 41 to 73 years (mean, 59.0 ± 9.56) are presented in Table 1. Most cases were advanced tumors. The incidence of perforated gastric cancer was 9.6% of all gastric carcinoma cases, and 4.2% of all gastric perforation cases. Four patients presented with sepsis before surgery. All patients underwent emergency surgery. Malignant gastric perforation was diagnosed subjectively during surgery in 11 (84.6%) patients. Among them one patient was diagnosed from intraoperative frozen section and was treated by palliative surgery due to his poor general condition. Two patients had preoperative diagnosis of gastric cancer. All cases were tumors that penetrate serosa (3 T3 and 10 T4), and all but one patient had metastatic lymph nodes. The disease was more frequently in stage III–IV (2–10 cases). Only one case of stage II (T3N0M0) gastric cancer was also observed. Surgical and Postsurgical survival data for patients with perforated gastric carcinoma are given in Table 2.

**Table 1:** Clinicopathological features of patients with perforated gastric cancer.

**Variable**	**Number of Patients**
**Age**	
Range (yr)/Mean	41–73/59
**Sex**	
Male	10/13
Female	3/13
**Preoperative diagnosis**	
Perforation	11/13
Cancer	2/13
**Location-Perforation side**	
Lower third	3/10
Middle third	5/10
Upper third	5/10
**Serosal invasion**	
Absent	0/13
Present	13/13
**Lymph node metastasis**	
Absent	1/13
Present	12/13
**Stage of disease**	
I	0/13
II	1/13
III	2/13
IV	10/13
**Surgery**	
Gastrectomy	7/13
Total	4/13
Subtotal	3/13
Local repair	6/13
**Lymph node dissection**	
Extended (D2, D3)	0/13
Limited (D0, D1)	7/13

**Table 2:** Postsurgical survival data for patients with perforated gastric carcinoma.

Case No	Age	Sex	PS	SI	LNM	TNM (cancer stage)	Type of surgery	DLND	PC	Survival (days)	Cause of death
1	50	M	(-)	(+)	(+)	T4N2M0-IV	Total gastrectomy, R1 resection	L	-	180	Primary cancer
2	67	F	(+)	(+)	(+)	T4N2M1-**IV**	Gastrostomi	_	-	3	Hospital mortality
3	58	M	(-)	(+)	(+)	T4N2M1-**IV**	Raphi+ Feeding jejunostomy	_	-	90	Primary cancer
4	51	F	(-)	(+)	(+)	T4N2M1-**IV**	Total Gastrectomy	L	-	7	Hospital mortality
5	53	M	(+)	(+)	(+)	T4N2M0-**IV**	Raphi	-	-	3	Cardiac arrest at the induction stage of anesthesia
6	41	M	(+)	(+)	(+)	T4N2M1-**IV**	Distal gastrectomy, R2 resection	L	-	1	Hospital mortality
7	65	M	(-)	(+)	(+)	T3N2M0-**III-B**	Total gastrectomy	L	-	90	Primary cancer
8	62	M	(+)	(+)	(+)	T3N1M0-**III-A**	Raphi	-	-	11	Cardiac arrest at the induction stage of anesthesia
9	65	M	(-)	(+)	(+)	T4N2M0-**IV**	Distal gastrectomy	L	Anastomotic leakage	22	Hospital mortality
10	52	M	(-)	(+)	(+)	T4N2M1-**IV**	Raphi	-	-	90	Primary cancer
11	57	F	(-)	(+)	(+)	T4N2M1-**IV**	Raphi	-	-	120	Primary cancer
12	73	M	(-)	(+)	(-)	T3N0M0-**II**	Distal gastrectomy	L	-	Since 09.03.2006	ALIVE
13	73	M	(-)	(+)	(+)	T4N1M0 **-IV**	Total gastrectomy	L	-	Since 23.04.2006	ALIVE

Abbreviations: PS:Presence of sepsis, SI:Serozal invazion, LNM: Lymph node metastas, DLND:Degree of lymph node dissection, PC:Postoperative compications, L:Limited.

Various surgical procedures, based on the subjective judgements of surgeons were performed. Poor general condition, extensive tumor spread, technical difficulties in resective procedures and severe peritonitis were the indications of the local repair. None of the patients had curative resection with radical lymph-node dissection.

Six (46%) patients with overt distal metastases or unresectable direct invasion were subjectively diagnosed as having incurable-nonresectable malignant gastric perforation or with their poor general condition and were treated by palliative surgery. (Simple closure or omental patch repair, and one case treated by tube gastrostomy via perforated area of the stomach). Of these patients, two had cardiac arrest at the induction stage of anesthesia due to their poor general condition; after the cardiopulmonary resusitation, these 2 patients had local repair and died at postoperative care unit.

Emergency total gastrectomy was performed in 4 patients (31%), and distal gastrectomy in 3 patients (23%). All 4 patients underwent total gastrectomy with limited lymph node dissection including perigastric lymph nodes only. Among this group, one patient had visceral metastases (hepatic), one had positive proximal resection margin and at the end of the surgery microscopic or macroscopic (R1 or R2) residual disease was left behind in all of the these patients.

Among the distal gastrectomy group, 2 patients underwent surgery with macroscopic residual disease (R2) and one without macroscopic residual disease but with only limited lymphadenectomy due to severe peritoneal inflamation. This patient was recommended to undergo a secondary surgery, aiming completness of lymphadenectomy but the patient refused to undergo revision surgery.

As a major surgical complication; an anastomotic leakage developed 8 days after surgery in a patient who underwent distal gastrectomy, and the patient died from anastomotic leakage and extensive tumoral dissemination 22 days later.

Six patients died within the 30 days postoperatively: two after distal and one after total gastrectomy, one after gastrostomy, and two after simple patch repair. The overall 30-day hospital mortality rate was 46%.

When patients were divided in to two groups according to have sepsis or not, it was found that having preoperative sepsis parameters was significantly correlated with 30-day mortality. The mean survival time was shorter in septic group compared to non-septic group (4.5 ± 4.43 days vs. 183.2 ± 200.4 days), the difference between both group was significant. (P < 0.01)

The overall survival time was 128.2 ± 184.8 days for all patients, it was 52.8 ± 52.9 days for locally treated group, and 192.9 ± 235.4 days for patients who underwent resectional surgery. The difference between the treatment groups was not significant. (P > 0.05)

## Discussion

Perforation of gastric adenocarcinoma is rare, and surgeons are unlikely to encounter more than a single case in their career. Patients with perforated gastric malignant neoplasms usually present with histories and symptoms that are not obviously different from those of the patients with benign gastroduodenal or other hollow viscus perforations [6]. Preoperative diagnosis of malignancy is unusual, accounting for about 30% of cases [1,3,6,11]. The only preoperative feature that may guide the surgeon is the age of the patient: Perforated gastric carcinoma usually occurs in elderly patients when compared with the patients with perforated peptic ulcers [6,12]. Similar differences in ages were observed by other authors [3,4,11,13]. Therefore, gastric perforation should raise suspicions of malignancy, particularly in elderly patients [7]. The mean age of the patients in our study is59.0 ± 9.56 years. This result is comperable to previous reports.

Preoperative diagnostic diffuculties encountered in gastric cancer perforation continue in the operation room [7,11]. Furthermore, during the emergency operation it is often impossible to confirm the diagnosis, particularly when a frozen section is unavailable [6,7]. Several studies have noted the description of 'intraoperative possible diagnosis of gastric cancer' for those cases [5,6,11]. The histopathological diagnosis was confirmed with intraoperative frozen section in only one case in the current study. The diagnosis of cancer of two cases were already made preoperatively. The diagnosis of the other 10 cases could be made with postoperative histopathological examination.

However, even if gastric cancer has been diagnosed pre- or perioperatively, it may still be difficult to assess the true extent of carcinoma and to determine local operability. Inflammatory changes associated with peritonitis have led the surgeon overestimate local tumor infiltration and the extent of lymph-node metastases intraoperatively [12]. Based on these fact, some authors recommended two-stage operation for PGC. In most instances the initial operation should, therefore, be directed at the treatment of perforation and peritonitis. After recovery of the patient and histological confirmation of malignancy, adequate staging can be completed and radical oncological operation for gastric cancer may be planned, if appropriate [12].

Lehnert et al. reported eight PGC patients eligible for radical surgery and two-stage operation approach had been performed with no operative mortality and good long-term success [12].

In contrast, in an analysis of 155 Japanese patients, Adachi et al. [1] demonstrated that 83% of patients underwent emergency gastrectomy with a mortality rate of 7% and 5-year survival rate of 40%. In a case report of perforation of advanced gastric carcinoma (with both serosal invasion and lymph node metastasis) who underwent emergency total gastrectomy with extended lymph node dissection Adachi et al. reported a survival of more than 7 years [13]. Similarly, Gertsch et al. reported the ratio of emergency gastrectomy as 88% with a mortality rate of 16%, and 6 patients were alive after a median time of 42 months [11]. With these results, they stressed that although many PGC were advanced tumors, an emergency gastrectomy should be the procedure of choise for the treatment. Although the decision on whether to submit patients to gastrectomy should be made on an individual basis, surgeon should be aware of that gastrectomy often offer the best curative or palliative option whenever technically feasible despite reported hospital mortality ranging between 23% and 42% [1,5,7,13]. In the present study, we performed one-stage gastrectomy in 7 cases. The mortality rate of the patients who underwent resectional surgery in the present study was 43%. This rate of mortality was somewhat higher than those results that reported by others Recently, Lee et al. reported 13 cases of PGC, all of them underwent gastric resection without maortality [4].

Roviello and his colleagues concluded that if a patient has a curable tumor and acceptable general condition, i.e. no sign of shock, localized peritonitis and no comorbidities, the treatment of choice seems to be radical total or subtotal gastrectomy with associated D2 or D3 lymphadenectomy or, for a less aggressive approach, two-staged radical gastrectomy. They also concluded that when general condition is good, but the tumor is at an advanced stage with no possibility of R0 resection, a palliative gastrectomy, if technically possible, is recommended considering the minor surgery-related mortality [6].

In the mentioned study, Roviello et al. also reported that; if a pathologist is not available and histologic examination is not possible during surgery, they suggest to perform a gastric resection, since for perforated peptic ulcer too the treatment of choice is resection both for the beter morbility and the lower rate of recurrence [6].

The present study included 12 cases of stage III or IV PGC patients, and only one case of stage II patient. This case of stage II PGC underwent emergency distal gastrectomy with DI lymph node dissection. After the recovery, the patient was offered a second surgery, aiming extended, completing lymhadenectomy, but patient refused this recommendation. The patient is still alive and disease-free. The other 12 patients underwent emergency gastrectomies (6 cases) with limited lymphadenectomy and incomplete (R1 or R2) resection; and local therapies (6 cases).

In our study, the ratio of locally treated patients was high (6 patients – 46%). The reason for this high ratio of local/non-resectional surgery was the high frequency of advanced stage disease.

All tumors treated with any form of local repair were at clinical stage IV of the disease; except for one, with stage III (T3NIM0). This patient had cardiac arrest during surgery and therefore, after resuscitation, he was treated by local surgery, and died in the postoperative period in the intensive care unit.

This high frequency of local/non-resectional surgery was also attributed to the poor condition of the patients, and difficulty in resection of the malignant tissue including perforation. Three surgery-related/hospital deaths were observed in 6 (50%) locally treated patients. This high ratio of hospital mortality in the locally-treated PGC cases was also observed by different authors. Roviello reported 4 cases of PGC who had been locally treated and died after surgery except for one case who died 5.2 months after the operation performed for the primary disease[6].

Malignant gastric perforation is a common manifestation of advanced cancer with serosal invasion and lymph node metastasis[4,6]. Nevertheless, as confirmed by various observations, gastric cancer can perforate even at an early stage [14]. Adachi et al.[1] analyzed the surgical results of 155 patients with PGC collected from the Japanese literature finding that there were 27 stage I tumors (19%), 16 stage II tumors (12%), 42 stage III tumors (30%), and 55 stage IV tumors (39%). Similarly, the present study contained solely one case of stage II patients and, 12 advanced stage PGC patients (92%).

Possible dissemination of tumor cells at the time of perforation of gastric carcinoma has been a matter of concern [1,12]. Malignant gastric perforation is commonly regarded as a sign of terminal disease, because it is thought to contribute to the peritoneal dissemination of cancer cells and early recurrence. Therefore, simple closure of the perforation has in the past been the preferred treatment method.

However, recent reports suggest that cancer perforation and peritoneal seeding do not necessarily influence survival in patients underwent gastrectomy [3-5,8]. The 5-year-survival rates of many series of patients with PGC were comparable with those of the patients with nonperforated gastric carcinoma [1,3,12]. In a study using multivariate analysis, tumor-node-metastasis stage was demonstrated as the only factor that was correlated with long-term survival in patients with PGC [11]. Nevertheless, there is a frequent belief that peritoneal contamination complicates the situation in patients with nutritional deficiency, and immun suppression, leading to sepsis and ileus, so that the patients get usually worse [1,11,13].

In their series, Gertsch et al.[11] also found that risk score (poor condition of the patients) was the only variable that was predictive of 30-day mortality among the factors such as patients age, the location of the tumor, the size of the tumor, depth of cancer invasion in the gastric wall and pTNM staging. Similarly Ozmen and colleagues, Kasakura and colleagues, and others all recently reported that, symptoms for a longer duration and the presence of pre-operative shock and other pre-operative complications were the significant factors predicting the hospital mortality [3,5,8].

We also found in the present study that the PGC cases who have had sepsis preoperatively, showed markedly increased rate of hospital mortality when compared to the cases without sepsis.

The rate of mortality due to surgery-related complications in patients with PGC who undergo emergency surgery has been reported to be as high as 11 to 16% [2,3,8]. The 30-day hospital mortality (46%) is seems very high in the present study. But all of this patients had seriously poor preoperative general conditions and advanced stages of their diseases.

This high hospital mortality ratio and unsuccessful outcames after PGC can be attributed to the poor condition of patients and failure to control sepsis [1,8].

In conclusion, perforation of gastric carcinoma is a serious complication that is observed mainly in advanced tumors. These patients had a poor prognosis because of the presence of advanced cancer and their poor general condition. It can be said that; thankfully, perforated gastric carcinoma is a rare condition.
